# CMP Pad Conditioning Using the High-Pressure Micro-Jet Method

**DOI:** 10.3390/mi14010200

**Published:** 2023-01-13

**Authors:** Xin Li, Yinggang Wang, Hongyu Chen, Wenhong Zhao, Qianfa Deng, Tengfei Yin, Suet To, Zhe Sun, Xi Shen, Wei Hang, Julong Yuan

**Affiliations:** 1 College of Mechanical Engineering, Zhejiang University of Technology, Hangzhou 310023, China; 2 Key Laboratory of Special Purpose Equipment and Advanced Processing Technology, Ministry of Education and Zhejiang Province, Zhejiang University of Technology, Hangzhou 310023, China; 3 State Key Laboratory of Ultra-precision Machining Technology, Department of Industrial and Systems Engineering, The Hong Kong Polytechnic University, Hung Hom, Kowloon, Hong Kong SAR, China; 4 School of Engineering, Zhejiang Agriculture and Forestry University, Hangzhou 310007, China

**Keywords:** chemical mechanical polishing, polishing pad, wear, conditioning, high-pressure micro-jet

## Abstract

In this study, in order to improve and restore the performance of the polishing pads and reduce the cost of chemical mechanical polishing, three types of material polishing pads, namely, polyurethane, damping cloth, and non-woven fabric, were selected for the experiment. Accordingly, each polishing pad was set up with diamond conditioner and high-pressure micro-jet (HPMJ) conditioning control experiments. Subsequently, the fluctuation ranges of the material removal rate on the three polishing pads were 2.73–3.75 μm/h, 1.38–1.99 μm/h, and 2.36–4.32 μm/h, respectively under the HPMJ conditioning method, while the fluctuation ranges of the material removal rate on the three polishing pads were 1.80–4.14 μm/h, 1.02–2.09 μm/h, and 1.78–5.88 μm/h under the diamond conditioning method. Comparing the polishing pad morphologies under SEM, we observed that the surface of the polishing pad after HPMJ conditioning was relatively clean, and the hole structure was not blocked. Contrastingly, there remained numerous abrasive particles on the surface after the conventional diamond conditioning and the hole structure was blocked. Thus, the HPMJ conditioning technology is better than the traditional diamond conditioning technology. Subsequently, the polishing pad after HPMJ conditioning has a longer service life and a more stable material removal rate than that after traditional diamond conditioning.

## 1. Introduction

Currently, chemical mechanical polishing (CMP) is considered the only effective processing method that can achieve comprehensive flattening and ultra-smooth and damage-free nanoscale surfaces.

There are many commonly used chemical mechanical planarization methods, including chemical mechanical polishing (CMP) [1,2,3], electrochemical polishing (ECMP) [4,5,6], ultrasonic-assisted polishing (UACMP) [7,8,9], etc. Song et al. [10] proposed a green polishing solution for 5083 aluminum alloy. When the polishing liquid contains H_3_Cit and does not contain H_2_O_2_, it will cause obvious corrosion defects on the surface of the aluminum alloy. When the polishing liquid contains H_3_Cit and H_2_O_2_, due to the oxidation of H_2_O_2_ on the aluminum alloy, the corrosion rate of the aluminum alloy in the process of chemical mechanical polishing is slowed down and the corrosion defects on the aluminum alloy surface disappear. Pang et al. [11] studied the ultra-precision CMP process of silicon carbide wafers; analyzed the effects of the volume flow of polishing liquid, rotating speed of polishing head, polishing pressure, and polishing time on the roughness; and determined the optimal process parameters as follows: the rotating speed of the polishing disc is 35 r/min, the rotating speed of the polishing head is 22 r/min, the polishing pressure is 250 g/cm^2^, the volume flow of the polishing liquid is 7.6 mL/min, and the polishing time is 8 h.

The polishing pads play an important role in CMP processing systems and the performances of different polishing pad types vary [12]. When a polishing pad is used for a certain period, its performance deteriorates, resulting in a decrease in the material removal rate (MRR) of the processed wafer and the appearance of scratches on the wafer surface [13,14]. Many scholars have carried out in-depth research regarding polishing pad conditioning. For example, Tsai [15] researched and designed a novel diamond conditioner, with which a more stable texture can be obtained after conditioning polishing pads. Yin et al. [16] investigated a combined diamond conditioner, which can meet the requirements of wafers that change different processes when performing CMP.

Although diamond conditioners have been used for most of the conditioning process, this method is extremely limited in slowing down the degradation in the polishing pad performance [17]. High-pressure water jet technology involves using tiny aperture nozzles to act on the workpiece surface through high-speed impact to achieve material removal, high-pressure cleaning, and rust removal [18]. This cutting technology is now mature and popularly used in industrial production [19].

The high-pressure microjet (HPMJ) method applies the high-pressure water jet technology to the polishing pad conditioning field. Many scholars worldwide have studied the HPMJ technique. For example, Lee et al. [20] compared the conditioning effect obtained with the HPMJ conditioning method with that achieved using the traditional conditioning method. The results showed that when using the HPMJ method to condition polishing pads, the same conditioning effect as the traditional conditioning can be obtained in half the processing time; moreover, the polishing pad conditioning efficiency and pad life are improved compared to the traditional method. SEIKE et al. [21] studied the effect of experimental parameters such as nozzle speed on the conditioning results. Moreover, they analyzed the kinetic energy range of water droplets ejected from the HPMJ system and its effect on the surface of the polishing pad. Their results demonstrated that the conditioning effect of the HPMJ system was related to the kinetic energy of the ejected droplets. Furthermore, the best conditioning effect is achieved when the nozzle angle is 25°, the fluid pressure is 10 MPa, and the distance between the nozzle and pad is 100 mm.

At present, many scholars at home and abroad have made some progress in polishing pads, but there are still some shortcomings. For example, the polishing pad self-conditioning technology still has the problem of “glaze” [22] in the late stage of CMP and the efficiency of CMP will gradually decline. Polishing pads are easily worn and their lives are reduced by diamond conditioner [23]. HPMJ technology can solve these problems effectively.

In this study, we examined the polishing pads manufactured using three different materials (polyurethane, damping cloth, and non-woven fabric), observed the MRR of traditional diamond conditioning and HPMJ conditioning wafer, and the surface morphology of the polishing pad. Subsequently, we provide theoretical guidance for HPMJ conditioning of polishing pads manufactured using different materials.

## 2. Materials and Methods

### 2.1. Materials

**Figure 1:**
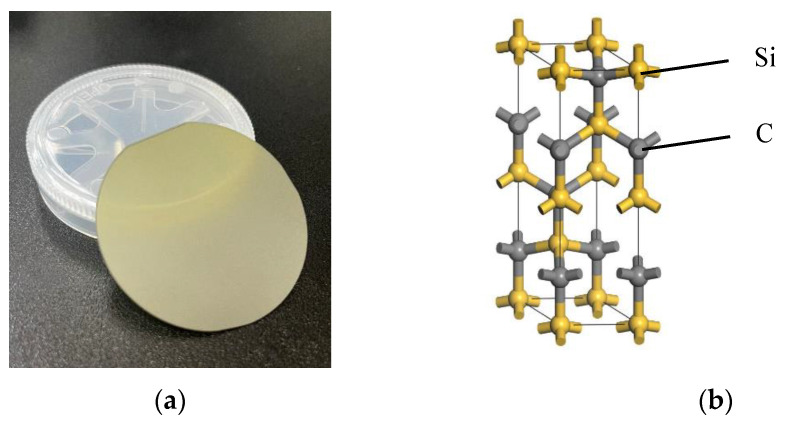
SiC Wafer and crystalline structure [24]. (**a**) 4H-SiC wafer; (**b**) 4H-SiC crystalline structure.

The sample used for the experiments was a 2-inch silicon carbide grinding wafer (diameter 50.8 ± 0.5 mm, thickness 400 ± 25 μm, model 4H-N); its physical appearance and crystalline structure are shown in Figure 1.

### 2.2. CMP Experiment

**Figure 2:**
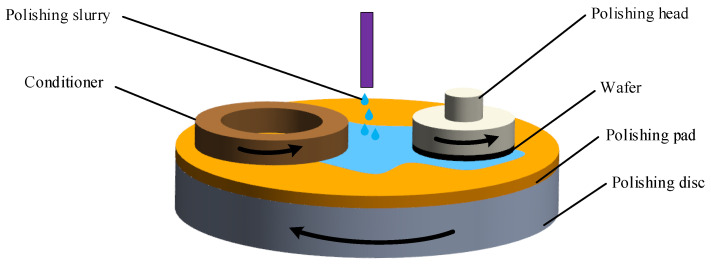
The schematic illustration of experimental device for CMP.

The silicon carbide wafer was glued to the polishing head with paraffin wax. The wafer was subjected to CMP processing on a plane-polishing machine (UNIPOL-1200S, Shenyang Kejing). A schematic of the CMP process is shown in Figure 2. The CMP polishing slurry consisted of diamond abrasive grains (HHM-B, 8000#), hydrochloric acid (HCl, 36% volume fraction) to adjust the pH, hydrogen peroxide (30% volume fraction) as the oxidant, and deionized water. According to the experimental parameters performed by other scholars [25], the CMP experimental parameters are listed in Table 1.

After polishing, the workpiece was ultrasonically cleaned with anhydrous ethanol for 5 min and subsequently dried using dust-free paper for subsequent surface quality and MRR inspection. The surface morphology of the SiC wafer was observed using a white-light interference profile meter and the roughness was measured using the Taylor roughness meter (TALYSUR I60). The five-point sampling method was used to ensure measurement accuracy. The sampling position is 1.5 mm from the center of the circle. The sampling length was 2 mm each time, the measurement speed was 0.5 mm/s, and the filter was selected ISO-2CR. A SARTOURIUS precision balance (precision, 0.01 mg) was used to weigh and calculate the MRR. The calculation formula is stated in Equation (1).

(1) $$MRR=\frac{M_{0}-M_{1}}{\rho \pi r^{2}t}$$

Here, *M*_0_ is the pre-experimental wafer mass, *M*_1_ is the post-experimental wafer mass, *ρ* is the wafer density, *r* is the wafer radius, and *t* is the processing time.

### 2.3. HPMJ Conditioning Experiment

**Figure 3:**
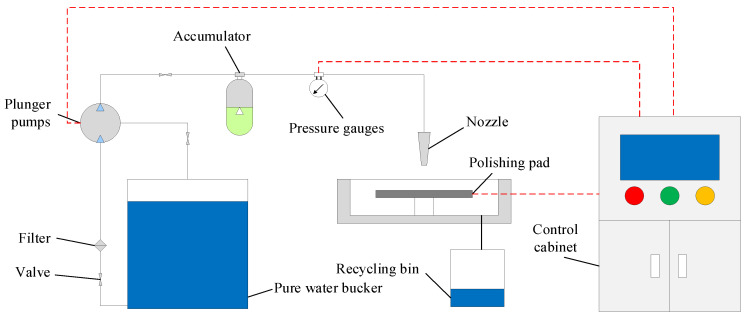
The schematic diagram of HPMJ conditioning system.

The polishing pad was placed on an HPMJ platform, as shown in Figure 3. The water flow pressure was controlled through the control cabinet. The water flow first passes through the filter to remove impurities in the water to prevent blockage of the nozzle and then through the plunger pump to allow the water flow to reach the experimentally required pressure value. An accumulator was used to stabilize the pressure in the system.

**Figure 4:**
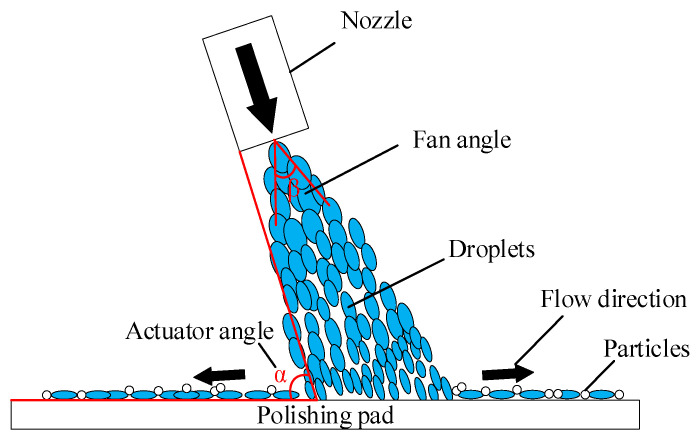
The schematic illustration of HPMJ conditioning.

Figure 4 shows the HPMJ conditioning mechanism, in which the special nozzle can turn the high-pressure water into discrete micro-droplets with high kinetic energy at the outlet. These droplets impact the surface of the polishing pad at high pressure and high speed, which can effectively clean the polishing pad surface and inside of the polishing pad without damaging its surface structure.

Assuming that water is an ideal fluid with continuous flow and incompressibility and referring to the Bernoulli equation, Equation (2) is used to calculate the jet velocity at the nozzle.

(2) $$\frac{P_{1}}{\rho_{1}}+\frac{V_{1}^{2}}{2}=\frac{P_{2}}{\rho_{2}}+\frac{V_{2}^{2}}{2}$$

Here, *P*_1_ denotes the pressure inside the nozzle, *P*_2_ denotes the pressure outside the nozzle, *V*_1_ denotes the average flow rate inside the nozzle, and *V*_2_ denotes the average flow rate outside the nozzle.

Applying the continuity equation between any two inner and outer sections of the nozzle yields Equation (3).

(3) $$\rho_{1}\cdot V_{1}\cdot A_{1}=\rho_{2}\cdot V_{2}\cdot A_{2}$$

The experimental conditioning parameters are listed in Table 2. After the conditioning, the surface morphology of the polishing pad was assessed and the hardness of the pad was measured using a Shore durometer. Subsequently, another CMP experiment was performed after the measurement to verify the experimental conjecture.

The nozzle used in this study is a hollow cylindrical nozzle. Considering its cross-sectional area of position $A=\pi \frac{d^{2}}{4}$, because the density of water inside and outside the nozzle is constant, and based on Equations (2) and (3), Equation (4) is obtained.

(4) $$V_{2}=\sqrt{\frac{2\left(P_{1}-P_{2}\right)}{\rho \left[1-{\left(\frac{d_{2}}{d_{1}}\right)}^{4}\right]}}$$

Because *P*_1_ >> *P*_2_, ${\left(\frac{d_{2}}{d_{1}}\right)}^{4}$ << 1, and *ρ* = 998 kg/m^3^, the theoretical initial velocity of the water jet can be simplified as stated in Equation (5).

(5) $$V_{t}=44.7\sqrt{P_{1}}$$

However, during the actual process, friction and other factors will cause energy losses in the jet, causing the actual initial velocity of the jet to be smaller than the theoretical initial velocity. Therefore, it is necessary to add the parameter κ. Accordingly, the actual initial velocity is provided by Equation (6).

(6) $$V_{w}=44.7\sqrt{P_{1}}\cdot \kappa$$

In the studies conducted by Momber [25] and Hashish [26], the range of *κ* is stated as 0.83–0.93. In this study, the middle value of 0.88 is selected according to the value range of jet pressure *κ*. The calculation yields *V_w_* = 78.7 m/s. The experimental parameters of the HPMJ conditioning are listed in Table 2.

## 3. Results and Discussion

### 3.1. Experimental Results

**Figure 5:**
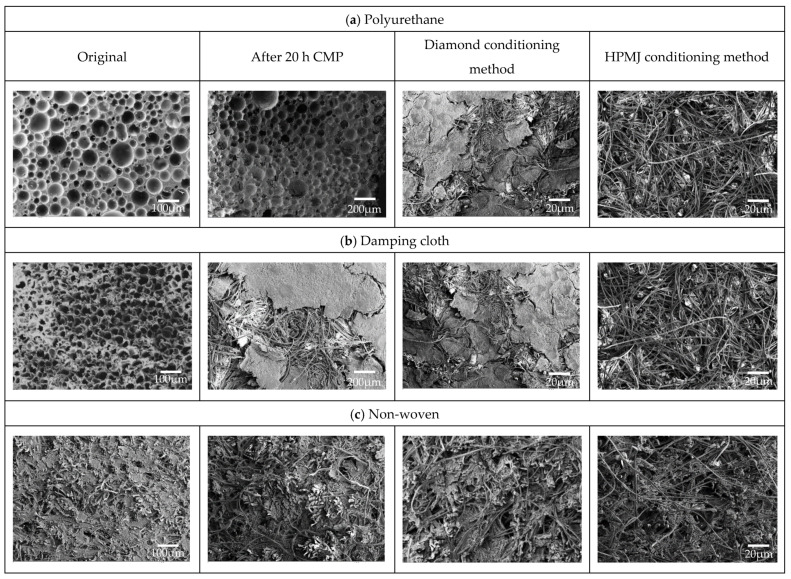
Comparison between surface morphologies of three polishing pads: (**a**) Polyurethane. (**b**) Damping cloth. (**c**) Non-woven.

**Figure 6:**
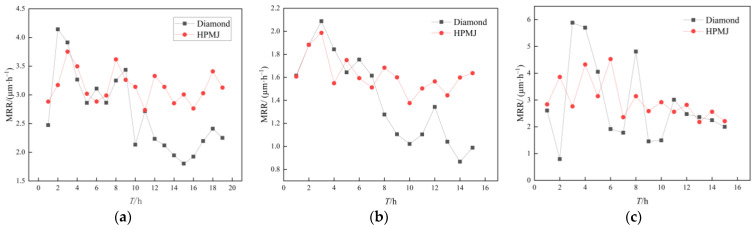
Variations in the MRR of three polishing pads processed using different conditioning methods: (**a**) Polyurethane. (**b**) Damping cloth. (**c**) Non-woven.

To investigate the difference between diamond and HPMJ conditioning approaches on the three polishing pads, a CMP control experiment was conducted using silicon carbide wafers. In the experiments, the control group was processed using a conventional diamond conditioner, while the experimental group was processed using the HPMJ conditioning. The surface morphologies of the three polishing pads were observed and analyzed using SEM before and after the CMP experiment, after the diamond conditioner and HPMJ conditioning. The results are shown in Figure 5. The MRR of the silicon carbide wafer processed with the two conditioning methods is shown in Figure 6.

### 3.2. Analysis of Results

Observe in Figure 6 that the MRR of the SiC wafers first showed an increasing trend and then a decreasing trend with increasing use time when the polyurethane polishing pad was used in the CMP experiments with the diamond conditioner; the overall removal rate was in the range of 1.80–4.14 μm/h. At a polishing time of 10 h, the removal rate was only 2.13 μm/h, indicating a decrease of 48.6%. The reason for the decrease in the removal rate is that most of the abrasive grains fill the holes of the polishing pad during the initial time and only a small portion of the grains are involved in the polishing process, resulting in a low wafer MRR. Subsequently, the holes of the polishing pad were filled with abrasive particles; the upper layer of abrasive particles that filled in the holes was involved in the removal process. At this time, polishing slurry dripping remained and additional abrasive particles were scattered on the surface of the polishing pad, increasing the wafer MRR. At a polishing time of 10 h, the removal rate was only half of the maximum value; accordingly, the wafer MRR tended to decrease as the experimental time increased, which can be attributed to the hardening of the polishing pad resulting from the polishing pad holes being completely filled with abrasive grains (the polishing pad hardness was measured before and after the experiment using a Shore durometer. The experimental result exhibited an initial hardness of 75 HD and hardness of 83 HD after 20 h), which decreased the polishing pad performance.

The overall MRR variation in the damping cloth polishing pads exhibited a decreasing trend in the CMP repeat experiments using diamond conditioners, within the range of 1.02–2.09 μm/h. When the polishing time was 10 h, the MRR of the wafer with diamond conditioning was 1.02 μm/h, corresponding to a reduction of 51.2%. This is because, after processing for a certain time, the polishing pad surface will be covered with an abrasive layer that cannot be removed by the diamond conditioner. The polishing pad’s ability to store the polishing slurry is reduced, causing it to be impossible for the added abrasive to enter the pad. The subsequent poor contact between the wafer and the polishing slurry leads to reduced chemical etching, which affects the polishing outcome. Simultaneously, the pore structure of the polishing pad was destroyed, the interior of the pad was clogged with abrasive particles, and the pad hardened. The hardness of the polishing pad was measured to be 65 HD, which was 61 HD before the experiment.

The MRR of the non-woven polishing pad was very unstable during the CMP repetition experiment with the diamond conditioner, reaching a maximum of 5.88 μm/h and a minimum of only 1.45 μm/h (the second-hour measurement result may be a measurement error, thus it is ignored). The surface holes were filled with abrasive particles and no obvious abrasive layer was formed. The reason is that the experiment indicated that the non-woven polishing pad structure strength was low, CMP process polishing pad wear was serious, and polishing pad debris constantly flaked off. Therefore, there was no formation of an abrasive layer, which requires a certain amount of time to accumulate, resulting in an unstable MRR. The hardness of the polishing pad was measured to be 89 HD, which was 87 HD before the experiment; in other words, a small increase in hardness was observed.

With regard to the trend of MRR in silicon carbide wafers after HPMJ conditioning in Figure 6, we observe that it is more stable compared to that obtained using a diamond conditioner.

The MRR of polyurethane polishing pad wafers was stable in the range of 2.73–3.75 μm/h with the HPMJ conditioning method. This is because the abrasive grains in the holes of the polishing pads were removed in time and the polishing process involved new abrasive grains. As observed from Figure 5, only a small amount of diamond abrasives remained in the holes of the polishing pad after HPMJ conditioning; accordingly, the polishing pad could be restored to its state before use. The hardness of the polishing pad was measured to be 75.5 HD, which was nearly the same as that before use.

The MRR of the damping cloth polishing pad wafers was stable in the range of 1.38–1.99 μm/h when using the HPMJ conditioning method. This is because there was no accumulated abrasive layer formed on the surface of the polishing pad and the ability of the polishing pad to store the polishing fluid remained unchanged, which stabilized the MRR. As shown in Figure 5, this method can effectively remove the abrasive layer on the surface of the polishing pad. The hardness of the polishing pad was 61.5 HD, which was nearly the same as that before use. Compared with the initial morphology, the surface of the polishing pad changed significantly. This is because the polishing pad surface loses its original morphology and reveals the internal polishing pad structure owing to constant wear and tear.

The MRR of wafers with non-woven polishing pads after using the HPMJ conditioning method exhibited a reduced fluctuation range compared to that achieved with diamond conditioning, with a maximum removal rate of 4.52 μm/h and a minimum of 2.17 μm/h. From Figure 5, it can be observed that the abrasive inside the hole of the polishing pad is cleaned but is different from the initial surface, which is caused by the continuous wear of the polishing pad during the CMP process. The hardness of the polishing pad after HPMJ conditioning was measured to be 86 HD using a Shore durometer, which denotes a reduction. The reason for this was that the polishing pad had more holes inside than on the surface; thus, the structure was softer.

**Figure 7:**
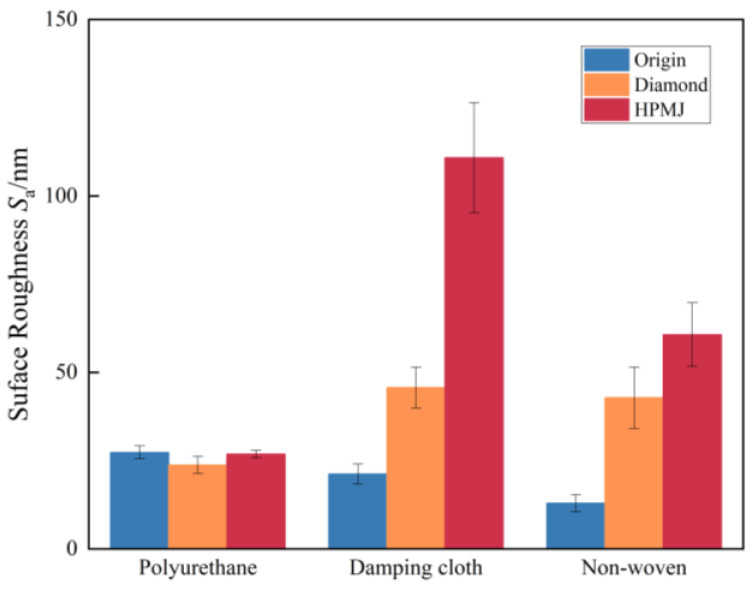
Surface roughness of three polishing pads after different conditioning procedures.

The polishing pads under the two conditioning methods were observed and analyzed using an ultra-deep-field microscope. A five-point sampling method was also used to measure the roughness of the polishing pad. Five points were randomly selected in the processing area of the polishing pad for measurement; finally, the average value was selected as the final value. The results are shown in Figure 7.

It can be observed from Figure 7 that the initial surface roughness *S_a_* of the polyurethane polishing pad was 27.33 nm and the *S_a_* value of the polishing pad surface conditioned with the diamond conditioner decreased to 23.75 nm after nearly 20 h of use. This is also because the holes in the polyurethane polishing pad are filled with abrasive grains, resulting in a flattening of the pad shape; the *S_a_* value of the polishing pad conditioned with HPMJ was 26.87 nm, which is very close to the initial surface roughness. The initial surface roughness *S_a_* of the damping cloth polishing pad was 21.23 nm and the *S_a_* value of the surface increased to 45.72 nm in the polishing pad conditioned with the diamond conditioner after nearly 15 h of use. The increase in roughness can be attributed to the destruction of the pore structure on the damping cloth surface and a disordered internal fiber structure, increasing the measured *S_a_* value, while the *S_a_* value of the polishing pad with HPMJ conditioning was 110.84 nm. This indicates that, although the polishing pad also reveals the internal fiber structure, the HPMJ conditioning method can clean up the residual abrasive particles on the surface to significantly improve the *S_a_* value.

The initial surface roughness *S_a_* value of the non-woven polishing pad was approximately 12.93 nm and the *S_a_* value of the polishing pad surface conditioned with the diamond conditioner increased to 42.84 nm after nearly 15 h of use. The reason for the increase in roughness is thought to be similar to the reasons stated for the damping cloth polishing pad. The *S_a_* value of the polishing pad conditioned with HPMJ was 60.71 nm and the *S_a_* value did not increase significantly because of the low structural strength of the non-woven polishing pad leading to constant flaking off during the polishing process, which resulted in few abrasive particles remaining on the surface. However, the HPMJ conditioning still had a certain effect. In general, all three polishing pads using two conditioning methods presented completely different results due to their different materials and structural strengths.

**Figure 8:**
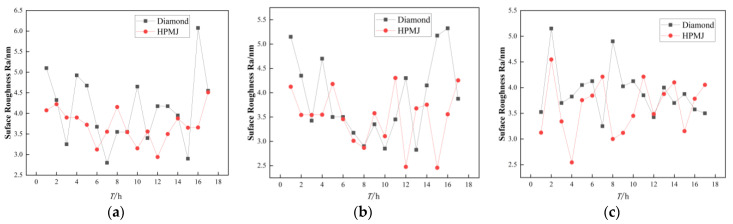
Wafer surface roughness of three polishing pad after different conditioning: (**a**) Polyurethane. (**b**) Damping cloth. (**c**) Non-woven.

Figure 8 shows the trend in the surface roughness on the wafers of the three polishing pads with two different conditioning methods.

For polyurethane polishing pads, the roughness values are more stable with HPMJ conditioning than with diamond conditioning, with an average roughness value of 4.10 nm for diamond conditioning and 3.71 nm for HPMJ conditioning, an improvement of 9.5%.

For the damping cloth and non-woven polishing pads, the two conditioning methods had little effect on the wafer roughness variation trends.

For the damping cloth polishing pads, the average roughness was 3.86 nm with diamond conditioning and 3.50 nm with HPMJ conditioning, corresponding to an improvement of 9.3%. For non-woven polishing pads, the average roughness with diamond conditioning was 3.89 nm and that with HPMJ conditioning was 3.62 nm, corresponding to an improvement of 6.9%.

To sum up, the roughness values are more stable with HPMJ conditioning than with diamond conditioning and the average roughness value with HPMJ conditioning is lower than that with diamond conditioning.

## 4. Conclusions

This study compares the differences between the two conditioning methods on three polishing pads. Overall, the HPMJ conditioning method can slow the MRR decline of the workpiece in CMP, prolong the service life of the polishing pad, reduce the cost of using CMP, and improve the wafer surface quality. The conclusions are as follows.

1. When using polyurethane polishing pad in the experiments, the MRR of wafers in the control group demonstrated an overall decreasing trend with increasing time. The removal rate of 2.13 μm/h was only 51.4% of the maximum value at 10 h of polishing time. HPMJ conditioning was performed every 3 h in the experimental group and the MRR of the wafer was always stable at 2.73–3.75 μm/h.
2. The removal rate trend of using the damping cloth polishing pad is similar to that of polyurethane, the MRR of wafers in the control group tended to decrease with increasing time. The removal rate of 1.02 μm/h was only 48.8% of the maximum value at 10 h of polishing time. HPMJ conditioning was performed every 3 h in the experimental group and the MRR of the wafer remained stable in the interval of 1.38–1.99 μm/h.
3. The experimental results obtained by using non-woven polishing pads are quite different from those obtained by the other two polishing pads, the MRR of wafers in the control group fluctuated significantly, between a maximum removal rate of 5.88 μm/h and a minimum removal rate of only 1.46 μm/h with no regularity. When the experimental group underwent HPMJ conditioning every 2 h, the MRR fluctuation of the wafer was relatively reduced to remain within the fluctuation interval of 2.36–4.52 μm/h for the first 7 h and 2.17–3.14 μm/h for the last 8 h, demonstrating an overall decreasing trend. This result provides a reference for further studies on high-pressure micro-jet and diamond conditioning for polishing pads.

## Figures and Tables

**Table 1:** CMP Experimental parameters.

Parameters	Values
Types of polishing pads	Polyurethane, Damping cloth, and non-woven polishing pad
Abrasive concentration (wt.%)	1
H_2_O_2_ (oxidizing agent) concentration (wt.%)	1
Polishing slurry pH	3~4
Base fluid	Ultra-pure water
Speed of polishing disc (rpm)	60
Speed of polishing head (rpm)	30
Pressure (kPa)	0.987 (Polyurethane), 0.493 (Damping cloth and non-woven polishing pad)
Time (h)	1

**Table 2:** HPMJ conditioning experimental parameters.

Parameters	Value
Water pressure (MPa)	2
Distance between nozzle and polishing pad (mm)	10
Nozzle actuator angle (°)	70
Nozzle lateral movement speed (mm/s)	1
Nozzle diameter (mm)	1.02
Time (min)	20
Nozzle fan angle (°)	30
Water jet speed(m/s)	78.7

## Data Availability

Data are only available upon request due to restrictions regarding, e.g., privacy and ethics. The data presented in this study are available from the corresponding author upon request. The data are not publicly available due to their relation to other ongoing research.
